# MachSMT: A Machine Learning-based Algorithm Selector for SMT Solvers

**DOI:** 10.1007/978-3-030-72013-1_16

**Published:** 2021-02-26

**Authors:** Joseph Scott, Aina Niemetz, Mathias Preiner, Saeed Nejati, Vijay Ganesh

**Affiliations:** 8 Eindhoven University of Technology, Eindhoven, The Netherlands; 9 Aalborg University, Aalborg East, Denmark; 10 University of Waterloo, Waterloo, Ontario Canada; 11 Stanford University, Stanford, USA

**Keywords:** SMT Solvers, Machine Learning, Algorithm Selection

## Abstract

In this paper, we present MachSMT, an algorithm selection tool for Satisfiability Modulo Theories (SMT) solvers. MachSMT supports the entirety of the SMT-LIB language. It employs machine learning (ML) methods to construct both empirical hardness models (EHMs) and pairwise ranking comparators (PWCs) over state-of-the-art SMT solvers. Given an SMT formula $\mathcal {I}$ as input, MachSMT leverages these learnt models to output a ranking of solvers based on predicted run time on the formula $\mathcal {I}$. We evaluate MachSMT on the solvers, benchmarks, and data obtained from SMT-COMP 2019 and 2020. We observe MachSMT frequently improves on competition winners, winning $54$ divisions outright and up to a $198.4$% improvement in PAR-2 score, notably in logics that have broad applications (e.g., BV, LIA, NRA, etc.) in verification, program analysis, and software engineering. The MachSMT tool is designed to be easily tuned and extended to any suitable solver application by users. MachSMT is not a replacement for SMT solvers by any means. Instead, it is a tool that enables users to leverage the collective strength of the diverse set of algorithms implemented as part of these sophisticated solvers.

### 1 Introduction

Satisfiability Modulo Theories (SMT) solvers are tools to decide the satisfiability of formulas over first-order theories such as bit-vectors, floating-point arithmetic, integers, reals, strings, arrays, and their combinations [44,9,24,18,47,20,46]. In recent years, SMT solvers have had a revolutionary impact on applications in software engineering (broadly construed), such as software testing [17,48] and verification [23,15,27,39], as well as in sub-fields of AI [53,35,30]. This impact is a driver for an insatiable demand for evermore efficient solvers, not only to scale to larger instances obtained from existing applications (e.g., automatic bug-finding

* This work was supported in part by DARPA (award no. FA8650-18-2-7861) and ONR (award no. N68335-17-C-0558).

in commercial software [26,4]), but also to solve problems from new application domains (e.g., verification and synthesis of cryptographic primitives [13]).

**Motivation for Algorithm Selection for SMT Solvers.** In response to this high demand, the SMT community has developed a plethora of solver heuristics and configurations. For example, in the 2019 edition of the annual SMT-COMP competition [10,31], more than 50 solvers and their configurations were submitted. Many of these solvers implement very different algorithms to tackle the satisfiability problem for (a combination of) first-order theories, with significantly varying performance profiles. For example, in the quantifier-free theory of floating-point arithmetic (QF_FP), there exist several substantially different decision procedures, e.g., bit-blasting [16], abstract CDCL [14], inter-reduction methods [55], and reduction to global optimization [22,11]. In this specific setting of floating-point solvers, input instances may be derived from a variety of applications, such as software verification or analysis of machine learning (ML) models [56]. In such a scenario, a very natural question arises: which solver or configuration is best for a given input instance?

Another well-known issue with many SMT solvers (even state-of-the-art ones) is that users may not know a priori which formula features or encoding would make an instance easy to solve. This can be very frustrating for users as they have to try a large number of different encodings with different solver configurations before they can figure out which combination works best for their specific scenario, which may result in a combinatorial explosion. Users have also noted that as their applications change, what was once a great solver configuration in an earlier setting is suddenly not very good in the newer one. One possible approach to address this problem is to use a portfolio of solvers, just as has been successfully done in the context of SAT solvers. Unfortunately, given the plethora of solvers (more than 50 in SMT-COMP 2019 and 2020) and configurations (CVC4 [9] alone utilizes 23 different configurations in a sequential portfolio setting for quantified logics) such an approach becomes quickly infeasible in the SMT solver setting.

**Brief Overview of MachSMT.** One way to address the above-mentioned problems is to use an automated algorithm-selection tool that can automatically and with high accuracy predict the best algorithm from a given set of algorithms for a specific input. Such a tool selects the best SMT solver from a set of solvers for a given SMT formula. To this end, we introduce MachSMT, a machine learning-based algorithm-selection tool. MachSMT supports the entirety of the SMT-LIB language [8]. It takes as input an instance for a specified theory of interest, and outputs a ranking of solvers predicted to have the lowest runtime. Internally, MachSMT is a set of machine learnt models constructed by analyzing the runtimes of solver configurations on benchmarks with respect to the frequencies of grammatical constructs (e.g., predicates, functions, rounding modes, etc.). Additionally, it defines other syntactical properties that can have influence in performance (e.g., quantifier nesting levels).

At a high-level, MachSMT works as follows. At its core, MachSMT uses two techniques to perform algorithm selection: empirical hardness models (EHMs)

and pairwise ranking comparators (PWCs). MachSMT uses frequencies of grammatical constructs from the SMT-LIB language [8], in addition to several other syntactical metrics for features pipelined with Principal Component Analysis (PCA) and AdaBoosting to construct its empirical hardness models and comparators.

An EHM for a given solver $\mathcal{S}$ is a mapping from an input instance $\mathcal{I}$ to a predicted runtime of $\mathcal{S}$ on $\mathcal{I}$. At runtime, given $\mathcal{I}$, MachSMT queries all EHMs for all solvers (that were considered during training) over $\mathcal{I}$, and outputs a ranking of solvers based on their predicted runtimes (top-ranked solver is predicted to solve the input problem the fastest). By contrast, a learnt pairwise ranking comparator (PWC) is a mapping that takes as input pair $(\mathcal{S}_1, \mathcal{S}_2)$ of solvers and an input instance $\mathcal{I}$, and outputs a ranking over the input solvers based on which one of them is predicted to have a lower runtime on $\mathcal{I}$ (denoted as $\mathcal{S}_1 \leq \mathcal{S}_2$ or $\mathcal{S}_1 \geq \mathcal{S}_2$). During evaluation, given an input instance $\mathcal{I}$, MachSMT uses the learnt PWC as a comparator to rank the set of solvers.

While algorithm selection has been considered in the broad setting of solvers (e.g., QBF solvers [50] and SAT solvers [67]) as well as certain specific SMT theories [57,5,64], we are not aware of previous work on algorithm selection aimed at the entirety of SMT-LIB [7]. Our results demonstrate that the MachSMT algorithm selector is highly effective, in that it outperforms the competition winners on the majority of tracks from the SMT-COMP in 2019 and 2020.

Perhaps the first algorithm selection tool in the context of logic solvers was SATZilla [67]. Since its introduction, SATZilla has had a tremendous impact on SAT solver research, winning multiple gold medals in the SAT competitions. Having said that, there are several significant differences between MachSMT and SATZilla. Briefly, SATZilla deploys a feature selection scheme to avoid the curse of dimensionality, while MachSMT leverages a learnt dimensionality reduction scheme, namely, Principal Component Analysis (PCA). In fact, a feature selection scheme would simply not scale in the context of SMT solvers given the very large number of learnt models that are incorporated into MachSMT. We discuss additional differences between SATZilla and MachSMT at length in Section 6.

It goes without saying that MachSMT is only as powerful as the underlying solvers that it has access to. MachSMT is clearly not a replacement for any particular SMT solver, but rather a tool that enables users to leverage the collective strength of the diverse set of algorithms and configurations implemented as part of these sophisticated solvers.

**Contributions.**

We make the following contributions in this paper.

1. **The MachSMT Algorithm Selection Tool**. We present the MachSMT tool, an algorithm selection tool for the entirety of SMT-LIB. MachSMT uses machine learning (ML) to construct EHMs and PWCs of solvers for algorithm selection. A key feature of MachSMT tool is that it is designed to be easily tuned and extended by SMT solver users (Section 3).

2. **Analysis of MachSMT over SMT-COMP 2019 and 2020 Benchmarks and Solvers**. We perform an extensive experimental analysis of MachSMT across all divisions from SMT-COMP 2019 and 2020. We observe that MachSMT improves on competition winners in 54 divisions, with up to 198.4% improvement in performance for the QF_BVFPLRA SQ '20 and up to 191.1% for the QF_BVFP SQ '20 division. We provide our learnt models, used in our experimentation, for ease of use and transparency. While building learnt models for MachSMT can be computationally expensive (a one time cost), installing, downloading, and using our models is easy (Section 4). All source code and learnt models from our experience can be found at: https://github.com/j29scott/MachSMT. The artifact is available at: https://zenodo.org/record/4458699.

The rest of this paper is structured as follows. Section 2 provides the necessary background, Section 3 gives a technical description of MachSMT, Section 4 gives an experimental evaluation of MachSMT over SMT-COMP 2019 and 2020, Section 5 provides an analysis of the experimental results, Section 6 describes related work, and Section 7 concludes the paper and discusses future work.

### 2 Background

In this section, we provide some background on algorithm selection via EHMs and PWCs, and the machine learning methods we use, such as principal component analysis (PCA) and k-fold cross validation.

#### 2.1 A Brief Overview of Algorithm Selection

The idea of algorithm selection was first proposed and formalized by Rice et. al. [51] in 1976. Researchers have long known that given a set of different algorithms and implementations for the same specification or problem, it is often the case that one of these implementations may perform poorly on a given class of inputs while another might perform very well. This is especially true for problems believed to be computationally hard (e.g., NP-hard). The reasons for this phenomenon could be as diverse as choice of data structures, fundamental differences between algorithms, or the fact that heuristics implemented as part of one algorithm can exploit the input problem structure or the underlying hardware better than the others.

It is natural to want to exploit the diversity in algorithmic approaches to minimize the cumulative runtimes. However, in practice users often deploy *greedy* algorithm selection – picking the best observed algorithm based on empirical analysis and testing. However, greedy algorithm selection can be sub-optimal when the best empirical algorithm has deficiencies relative to other algorithms on certain families of inputs.

With the recent advances in AI and ML, researchers are beginning to leverage these new technologies to advance algorithm selection. To the best of our knowledge, there are two key approaches for ML-driven algorithm selection in the context of constraint solvers: through the use of Empirical Hardness Models (EHMs), and through Pairwise Ranking Comparators (PWCs).

**Algorithm Selection via Empirical Hardness Models (EHMs):** Let $\mathcal{I}$ be an input in the language of $\mathcal{S}$ with a corresponding feature vector $\vec{x} \in \mathbb{R}^n$. For an algorithm $s \in \mathcal{S}$, an EHM is a learnt function $f_s : \mathbb{R}^n \to \mathbb{R}$ that predicts the runtime of $s$ on $\mathcal{I}$. An EHM is constructed with an ML regression model trained on collected runtime data. The algorithm is then selected by computing:

$$\underset{s \in \mathcal{S}}{\operatorname{argmin}} f_s(\vec{x})$$

**Algorithm Selection via Pairwise Ranking Comparators (PWCs).** Let $P$ be the set of all unique pair sets (sets of size two). For each $p = (\mathcal{S}_i, \mathcal{S}_j) \in P$, construct a learnt comparator $f_p : \mathbb{R}^n \to \{0, 1\}$, that returns 0 if algorithm $\mathcal{S}_i$ solves $\mathcal{I}$ faster than $\mathcal{S}_j$, and 1 otherwise. For an input $\mathcal{I}$ with a feature vector $\vec{x}$, we compute a ranking of algorithms as a map $r$ over $\mathcal{S}$, where for $s \in \mathcal{S}$, $r[s]$ is the ranking of solver $s$ that represents: "how many solvers in $\mathcal{S}$ are faster than $s$ in solving the input $\mathcal{S}$", or more formally: $r[s] = \Sigma_{p:s \in p} f_s(\vec{x})$. The selected solver is then the minimum ranked solver, i.e.,

$$\underset{s \in \mathcal{S}}{\operatorname{argmin}}\ r[s]$$

#### 2.2 Supervised Learning, Adaptive Boosting, Curse of Dimensionality, and K-Fold Cross-Validation

Supervised learning is one of the most predominant areas of ML. Supervised learning takes as input a dataset of features $X$ and labels $Y$, and each datapoint $\vec{x} \in X$ has a label $y \in Y$. A datapoint is a real valued vector $\vec{x} \in \mathbb{R}^n$ describing a sample. The learning problem is said to be a *classification* problem if the labels $y \in Y$ come from a fixed and finite set of classes $\mathcal{C}$ (e.g., a set of algorithms). Alternatively, the learning problem is a *regression* problem if the labels are real valued (e.g., runtimes).

One efficient and effective approach to supervised learning is *Adaptive Boosting (AdaBoost)*. AdaBoost is an *ensemble* approach to machine learning invented by Freund and Schapire et. al. [21], which won the Gödel Prize in 2003. In ensemble learning, a set of learning algorithms (e.g., weak learners) are trained, and predictions are made diplomatically across the set. In this paper, we exclusively consider AdaBoost to solve both the classification and regression problems for algorithm selection. We use an ensemble of 200 decision trees in the AdaBoost algorithm. For more, we refer to Drucker et al. [19].

While supervised learning has had tremendous impacts in several areas of research, there are pitfalls, such as the *curse of dimensionality* (CoD). Consider the convex polytope $P$ formed around the convex hull of $X$. The volume of $P$

increases exponentially with the dimensionality of $X$ requiring an exponential amount of datapoints to avoid extreme sparsity in $X$. Sparsity in datasets is one of the leading causes of poor performances in learnt models [28]. There is a large literature on managing the CoD. In this paper, we discuss *feature selection* and deploy *dimensionality reduction* solutions. In feature selection, a new dataset $X'$ is computed from $X$ by selecting the subset of features that are the most performant on a validation dataset. Feature selection was deployed in the successful SATZilla algorithm selection tool for Boolean satisfiability.

Despite the success of feature selection in SATZilla, feature selection does have some flaws. First, there is a significant loss of information. In the case of SATZilla, a feature vector composed of more than a hundred values describing an input is reduced to just five values. Second, the total number of feature subsets is exponential in the number of features. While there has been a great deal of research in reducing the time spent searching for high performing subsets [65,36], in our experiments, we found it to be the most computationally taxing component of the SATZilla framework.

When evaluating the performance of a supervised learning model, a training set is used to construct the learnt model and a testing set is set aside to evaluate. However, this method alone can be prone to overfitting and selection bias [54,43]. Instead, researchers often use $k-$fold cross-validation to evaluate their learnt models. In $k-$fold cross validation, the dataset is split into $k$ sets, and the learnt model is trained on $k-1$ sets and is evaluated on the set that is left out. This process is repeated $k$ times so each set gets evaluated.

#### 2.3 Unsupervised Learning and Principal Component Analysis

Unsupervised learning, in contrast to supervised learning, is the study of detecting patterns in an unlabelled dataset $X$. Applications of unsupervised learning include dimensionality reduction [66,63], clustering [29,72], and anomaly detection [38,1]. Principal Component Analysis (PCA) is an unsupervised learning dimensionality reduction technique. PCA computes an orthogonal transformation of a dataset $X$ composed of points in $\mathbb{R}^n$ to a new data set $X'$ composed of points in $\mathbb{R}^{n'}$ where $n' < n$. PCA is an incremental algorithm, wherein, each iteration a new component (or dimension) is computed. On the first iteration, a hyperplane is fit around the dataset $X$ and its corresponding spanning vector is the first element of the basis around the transformation onto $X'$. On each subsequent iteration, a new hyperplane is computed under the additional constraint of it being orthogonal to its predecessors. This process is repeated until the desired number of iterations is achieved [32,66].

### 3 An overview of MachSMT

In this section, we provide an overview of the MachSMT tool. The architecture diagram of MachSMT is presented in Figure 1.

| Feature ID | Description |
|---|---|
| 1–4 | Frequency of problem description grammatical constructs (e.g., assert, check-sat, etc.) |
| 5–13 | Frequency of declaration/definition grammatical constructs (e.g., declare-const, define-fun, declare-sort, etc.) |
| 14–15 | Frequency of the echo/exit grammatical constructs |
| 16–27 | Frequency of the get-* grammatical constructs (e.g., get-model, get-unsat-core, etc.) |
| 28–29 | Frequency of the push/pop incremental benchmark grammatical constructs |
| 30–31 | Frequency of the reset/reset-assertions grammatical constructs |
| 32–35 | Frequency of the set-* grammatical constructs (e.g., set-logic) |
| 36–37 | Frequency of the forall/exists quantifiers |
| 38 | Frequency of let bindings |
| 39–49 | Frequency of core/Boolean constructs, sorts, and literals (e.g., true, Bool, and, =>, ite, distinct, etc.) |
| 50–52 | Frequency of grammatical constructs of the theory of arrays (e.g., select, store, etc.) |
| 53–88 | Frequency of grammatical constructs of the theory of bit-vectors (e.g., BitVec, bvor, bvuge, bvsge, bvult, etc.) |
| 89–135 | Frequency of grammatical constructs of the theory of floating-point (e.g., fp.add, Float32, RNE, fp.eq, fp.isNaN, fp.to_real, etc.) |
| 135–150 | Frequency of grammatical constructs of the theory of integers and reals (e.g., Int, Real, $*$, $+$, to_real, is_int) |
| 151 | Average number of selects per array |
| 152 | Average store chain depth per array |
| 153–155 | Average/Median/Deviation of BV adder chains |
| 156–158 | Number of forall/exists variables and their ratio |
| 159 | Average quantifier nesting level |
| 160–161 | Average arity and applications of uninterpreted functions |
| 162 | Size of the smt2 file in bytes |

Table 1: Complete list of the 162 features used in MachSMT

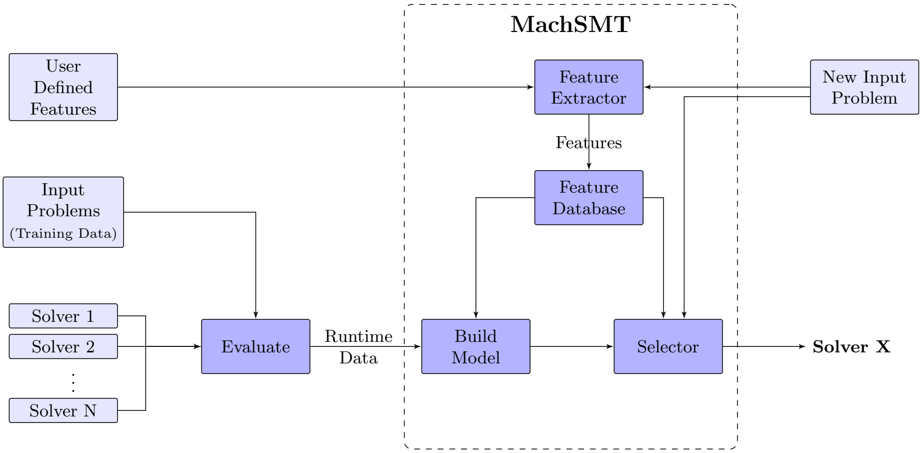

**Fig. 1** *Architecture of MachSMT.*

#### 3.1 Features, Preprocessing, and Learning

MachSMT uses a feature vector with 162 entries (i.e., dimensions). A complete description of each feature is provided in Table 1. We deploy two strategies to mitigate taxing feature calculation times, which can severely impair algorithm selection solutions. First, all features are entirely syntactical properties of the input. This is a major difference between MachSMT and other algorithm selection solutions, such as SATZilla. Second, all features are calculated within a strict and user-adjustable timeout (default 10s). On a timeout, the feature value is recorded as $-1.0$.

MachSMT performs three key preprocessing steps before constructing any learnt models over a given dataset. We describe each subsequently. First, all feature values are scaled to zero mean and unit variance[3]. This data normalization technique is common in ML research and applications to improve both model efficiency and numerical robustness. The second step in the preprocessing pipeline is computing the polynomial interaction terms of degree two on the resultant normalized feature vector. These polynomial features make interacting correlations of features explicit. These first two preprocessing steps are included in the SATZilla preprocessing pipeline [71].

As discussed in Section 2, ML in a high dimensional space is prone to the curse of dimensionality. While other algorithm selection solutions (e.g., SATZilla) commonly implement feature selection solutions, we propose the use of learnt dimensionality, namely PCA. As discussed above, feature selection can be a proactive solution to the curse of dimensionality but presents many challenges when applying to SMT. Internally MachSMT manages more than a thousand learnt models, and calculating optimal feature subsets for each one is infeasible.

[3] $\frac{x-\mu}{\sigma}$, where $x$ is a feature sample, $\mu$ is the mean across the specific feature on the training set, and $\sigma$ is the deviation across the specific feature on the training set.

The third and final preprocessing step is applying PCA on the resultant polynomial features. The final feature vector is composed of the first 35 principal components. PCA is the final step in the MachSMT preprocessing pipeline. The resultant feature set is used when constructing the learnt models with AdaBoost. We use AdaBoost for both regression in the EHMs and classifications in the PWCs. We configure AdaBoost with 200 decision tree estimators and linear loss. MachSMT uses scikit-learn and numpy as its ML backend and the entire tool is written in Python [49]. MachSMT is easily extensible and supports any ML model/pipeline under scikit-learn syntax.

#### 3.2 Variants of MachSMT

MachSMT implements the following algorithm selection solutions.

1. **MachSMT-SolverEHM** – This variant of MachSMT is analogous to the algorithm selection approach taken by SATZilla. As described in Section 2, an EHM is constructed for each solver, and the selected solver is computed by taking an argmin over all predictions.
2. **MachSMT-SolverLogicEHM** – This approach is similar to MachSMT-SolverEHM, with the key difference being an EHM is constructed for every solver, logic pair. As state-of-the-art SMT solvers implement significantly different algorithms depending on the logic of the input problem, datapoints from different logics could negatively skew predictions.
3. **MachSMT-SolverPWC** – This variant of MachSMT deploys the PairWise comparator approach as described in Section 2. In this variant of the PWC, comparators are trained for every pair of solvers across all provided data.
4. **MachSMT-SolverLogicPWC** – This variant of MachSMT is analogous to MachSMT-SolverPWC, with the key difference that solver-wise comparators are constructed by only training on the benchmarks of a common logic.

MachSMT by default creates models for all aforementioned approaches to algorithm selection. In evaluation, MachSMT evaluates each approach's performance on each logic. In deployment, MachSMT uses the approach that had the best-observed performance in evaluation.

#### 3.3 Using MachSMT

MachSMT consists of three core tools, which are used to build, evaluate, and deploy MachSMT, respectively.

1. `machsmt_build` – This tool is the interface for building MachSMT's database around the solvers and benchmarks provided by the user. It takes as input a csv data file denoting the columns 'solver', 'benchmark', and 'score'. The output is a library directory containing the resultant database, and learnt models under default settings.

```
machsmt_build -f data.csv -l /path/to/lib/dir
```

| Logic, Track, Year | Winner | Improvement over Random [%] | Improvement over Winner [%] | Distance from VBS [%] |
|---|---|---|---|---|
| QF_BVFP, SQ'20 | Bitwuzla | 195.1 | 191.1 | 86.2 |
| QF_BVFPLRA, SQ'20 | MathSAT5 | 199.1 | 198.4 | 34.0 |
| QF_UFBV, SQ'19 | Yices | 153.5 | 113.3 | 95.3 |
| NRA, SQ'19 | Vampire | 169.6 | 114.0 | 99.2 |
| QF_NRA, SQ'19 | Yices | 101.3 | 71.5 | 52.1 |
| QF_UFNRA, SQ'19 | Yices | 148.1 | 77.1 | 36.1 |
| QF_LIA, SQ'20 | MathSAT5 | 132.6 | 71.5 | 46.4 |
| QF_UFBV, SQ'20 | Yices | 137.8 | 67.4 | 109.4 |
| QF_UFNRA, SQ'20 | Yices | 151.3 | 47.9 | 42.6 |
| QF_ABV, INC'20 | Yices | 169.4 | 50.8 | 114.6 |
| QF_NRA, SQ'20 | Yices | 82.5 | 41.2 | 46.5 |
| QF_AUFLIA, SQ'20 | Yices | 200.0 | 37.2 | 27.9 |
| BV, SQ'20 | CVC4 | 112.1 | 30.6 | 117.8 |
| QF_LRA, SQ'19 | SPASS-SATT | 89.3 | 28.4 | 59.5 |
| QF_UFLRA, INC'20 | Z3 | 133.3 | 26.2 | 19.9 |
| QF_ANIA, SQ'20 | MathSAT5 | 199.0 | 26.1 | 61.6 |
| QF_LIA, SQ'19 | SPASS-SATT | 161.5 | 29.8 | 66.3 |
| BV, SQ'19 | Q3B | 91.8 | 25.0 | 83.7 |
| LIA, SQ'20 | CVC4 | 172.5 | 22.3 | 19.6 |
| QF_UFNIA, SQ'20 | CVC4 | 125.6 | 21.9 | 105.0 |
| UFDTNIRA, SQ'20 | Vampire | 123.9 | 24.0 | 92.6 |
| QF_UFLRA, INC'19 | Z3 | 110.0 | 19.6 | 22.0 |
| QF_FP, SQ'19 | COLIBRI | 41.6 | 18.4 | 62.8 |
| QF_AUFBV, SQ'20 | Yices | 82.0 | 20.4 | 3.6 |

Table 2: Selected results of MachSMT on data from SMT-COMP 2019 and 2020. All numbers are percent differences of PAR-2 scores across all benchmarks. Columns 3 and 4 show the improvement over random selection and competition winners (higher is better). Column 5 shows the PAR-2 difference to the VBS (lower is better).

2. `machsmt_eval` – This tool takes as input the library directory generated by `machsmt_build` and evaluates it under k-fold cross validation and provides a summary of results. It further tunes MachSMT to use the best empirically observed variant based on the logic and track of the input benchmark.

```
machsmt_eval -l /path/to/lib/dir
```

3. `machsmt` – This tool is the primary interface to MachSMT' algorithm selection. Provided an input benchmark and its library files, it will output a ranking of solvers that are predicted to solve the benchmark the fastest.

```
machsmt benchmark.smt2 -l /path/to/lib/dir
```

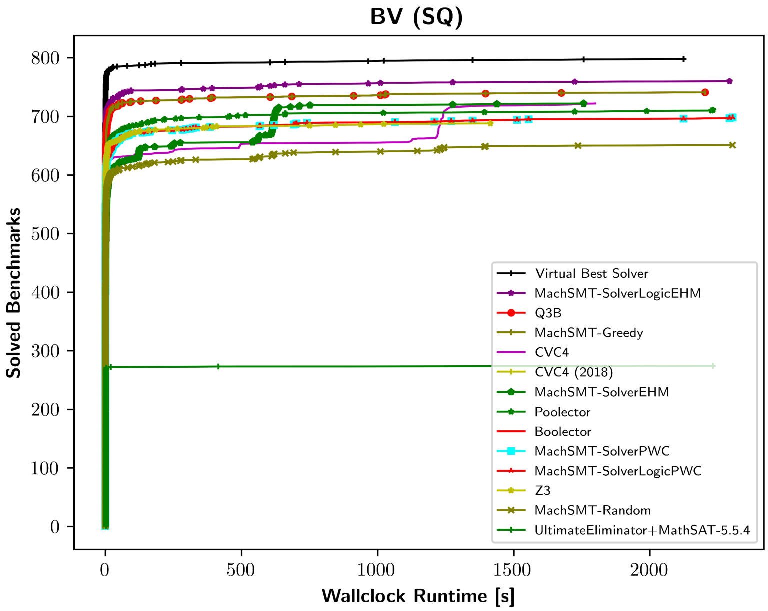

**Fig. 2** *Plot for BV in the Single Query (SQ) Track in SMT-COMP '19.*

#### 3.4 User-defined Features

We include a simple interface for users to extend the considered features in MachSMT's algorithm selection. All that is required is to create a Python method that returns a single floating-point number (or an iterable object thereof) representing the feature. As input, the user enters the path of the SMT-LIB input, as well as its logic and track. If a user feature is to be considered by MachSMT, the user-defined procedure should return its floating-point representation; otherwise, it returns none. All user-defined features are automatically included in building MachSMT. These custom features in principal can significantly affect the accuracy of MachSMT when engineered to target a specific class of benchmarks.

### 4 Experimental Evaluation of MachSMT on SMT-COMP 2019 and 2020 Data

In this section, we present the evaluation of our MachSMT tool (refer to Table 2 and CDF plots in Figures 2–6), specifically with the benchmarks, solvers, and solver runtime analysis from SMT-COMP 2019 and 2020. The artifact is available at: https://zenodo.org/record/4458699.

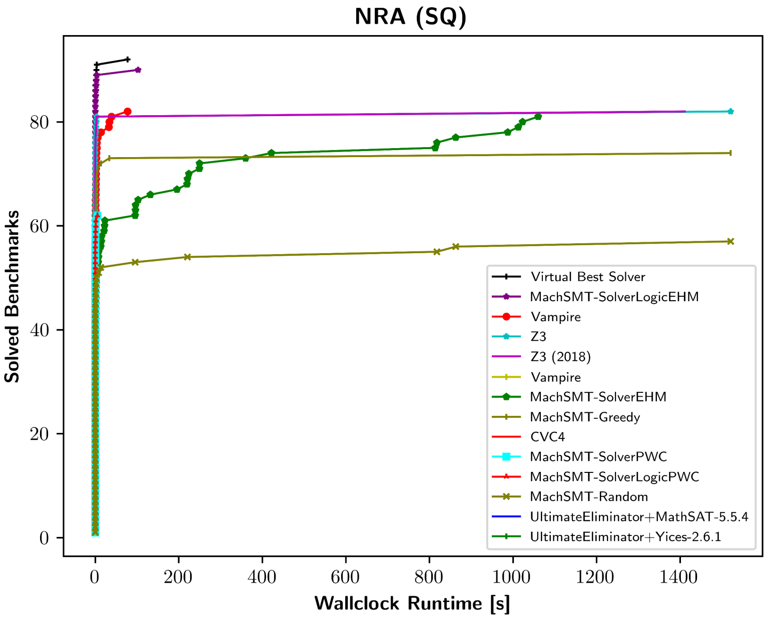

**Fig. 3** *Plot for NRA in the Single Query (SQ) Track in SMT-COMP '19.*

#### 4.1 Experimental Setup and Methodology

In this experiment, we used the benchmarks, timing analysis, and solvers provided by the organizers of the SMT-COMP 2019 and 2020 competitions [31,6]. In both years, all solver input queries were performed on the StarExec computing service [58], which consists of a cluster of 2.4 GHz Intel Xeon machines running Red Hat Enterprise Linux 7.2. Each solver/benchmark pair was configured to have 4 cores and 60GB of memory available. The time limit for each pair was 2400 seconds in 2019, and 1200 seconds in 2020.

We evaluate MachSMT and all of its variants using $k$-fold cross validation (with $k = 5$). In cross validation, the dataset is randomly partitioned into $k$ subsets per division. A model is then trained over $k-1$ subsets and makes predictions over the subset that is excluded from training. This process is repeated to obtain fair predictions for each subset. Cross validation is commonly deployed to analyze machine learning models. For more details, please see Section 2.

#### 4.2 Experimental Results

For every division, we evaluated MachSMT by checking whether we beat the competition winner from each division. For the sequential tracks, we evaluate solvers across, according to PAR-2 scores (i.e., the wallclock runtime on success-

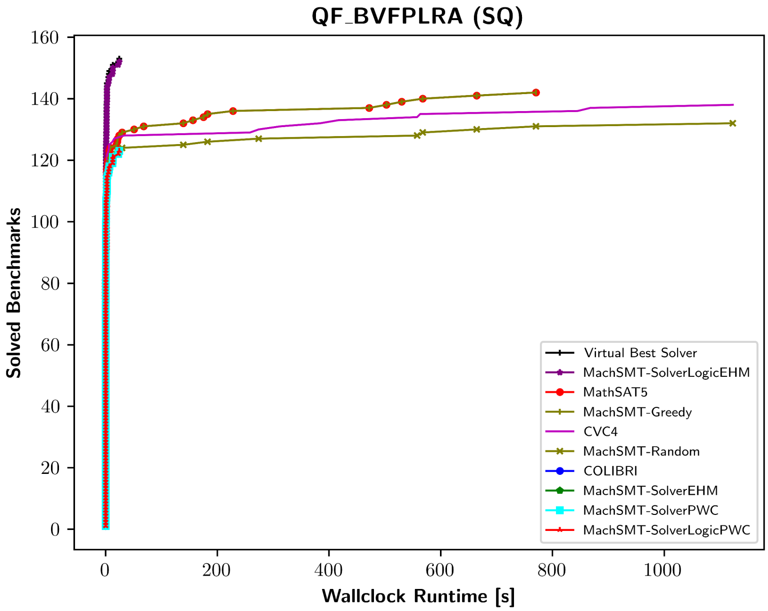

**Fig. 4** *Division QF_BVFPLRA in the Single Query Track in SMT-COMP 2020.*

ful termination, otherwise twice the wallclock timeout)[4] [42]. For incremental tracks, we use the following formula:

$$w + (2 * t/n) * (n - m)$$

where $w$ is the wall clock runtime, $t$ is the wallclock timeout, $n$ is the total number of check-sats in the benchmark, and $m$ is the total number of check-sats successfully solved.

We present select results in Table 2. We consider three baselines when evaluating MachSMT, namely: random algorithm selection, the competition winner, and the virtual best solver (VBS) (note, VBS is perfect algorithm selection and cannot be beaten). We consider all divisions of at least 25 benchmarks and observe MachSMT to improve on the competition winner in 54 out of 85. We report the results for MachSMT-SolverLogicEHM in the table as it is by far the most performant, dominating in all divisions except for 4.

We present select CDF plots in Figures 2-6. A CDF plot is a visualization of how a solver performs on a database of inputs. A point (X,Y) denotes that a solver $\mathcal{S}$ solves $Y$ inputs within $X$ seconds each.

[4] In case of an incorrect answer, the score is recorded as 10 times the wallclock timeout.

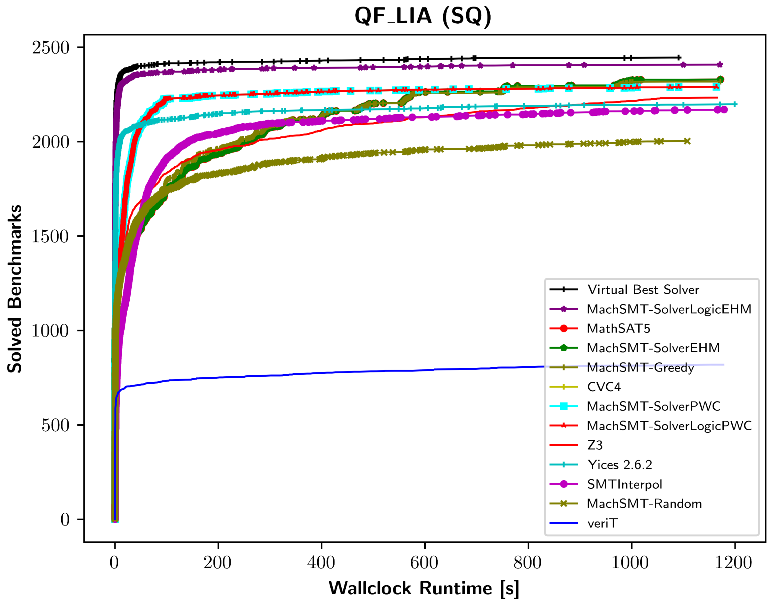

**Fig. 5** *Division QF_LIA in the Single Query Track in SMT-COMP 2020.*

### 5 Analysis and Discussion of Results

In Section 3.2, we describe four formulations of MachSMT. In our evaluation (see Table 2), we observe MachSMT-SolverLogicEHM to be significantly more performant than all other formulations. When evaluating over SMT-COMP, in all divisions that MachSMT improved over the competition winner, MachSMT-SolverLogicEHM was the most performant in all except for three (which were won by MachSMT-SolverLogicPWC).

Our experimental results validate the idea that algorithm selection (in particular through the use of EHMs) can be a powerful way to address the combinatorial explosion that solver users face when trying to decide which solver-configuration pair is best suited for their application. We note that MachSMT is particularly powerful in the context of logics, such as QF_UFBV, that are derived from a diverse set of applications and a wide variety of algorithms have been designed to solve them. As has been noted in previous work, algorithm selection methods work well for non-homogeneous benchmarks, especially where there is no single algorithm (solver) that performs the best across the board. EHMs are an effective way to distinguish between such algorithms given a problem instance and predict which one might perform the best on said instance.

One major threat to the validity of any ML solution is the generalizability of the learnt models on unseen data. It has been noted in previous work that a practical way to address this issue is to use $k-$fold cross validation scheme [54,43], thus motivating our use of this approach in our experiments. We further note

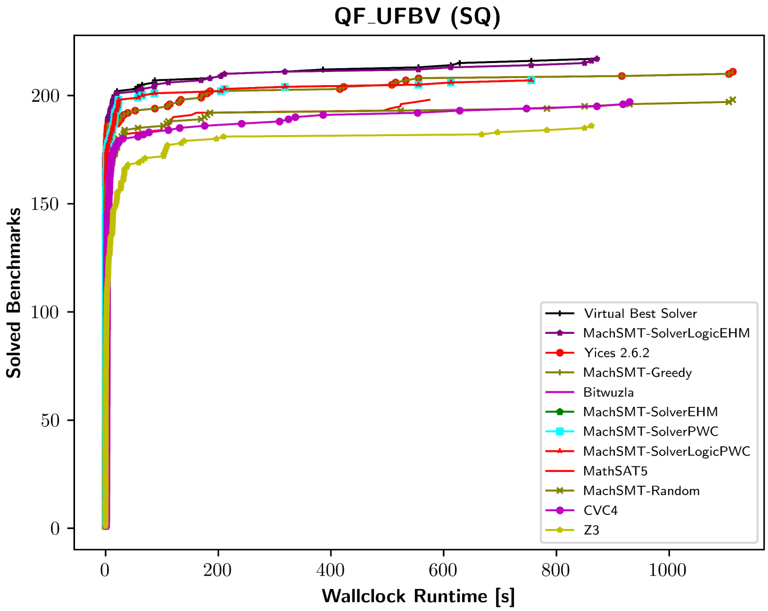

**Fig. 6** *Division QF_UFBV in the Single Query Track in SMT-COMP 2020.*

that our evaluation of MachSMT includes decades of runtime analysis and more than 100 GB of benchmarks spanning numerous applications, giving us greater confidence in the robustness of our results.

## 6 Related Work

In this section we provide an overview of previous work on algorithm selection in the context of constraint solvers and contrast it with MachSMT.

### 6.1 Key differences between SATZilla and MachSMT

As mentioned above, SATZilla was the first algorithm selection method in the context of logic solvers [67]. While our work is inspired by SATZilla, MachSMT differs from SATZilla in several key ways. First, SATZilla deploys a feature selection scheme to avoid the curse of dimensionality. While good in practice for the SAT setting, feature selection does lose significant amounts of information. Further, it can be very expensive to compute optimal feature subsets.

By contrast, MachSMT leverages a learnt dimensionality reduction scheme, namely, Principal Component Analysis (PCA). The key advantage of PCA is that it does not perform a search for optimal feature subset (like one has to do in the context of feature selection), and hence is significantly more efficient. In fact, a feature selection method is unlikely to scale for SMT solvers, unlike SAT, simply because of the significantly larger number of features, logics, and solvers that one has to contend with. Second, MachSMT deploys a modern ML pipeline, including an ensemble learning approach, namely Adaptive Boosting [21].

#### 6.2 Algorithm Selection for Logic Solvers and Their Applications

Algorithm selection tools have a rich history and have been around since at least 1976 when Rice et al. were the first to propose it [51]. Algorithm selectors have been extensively used in many contexts, e.g., classifiers for machine learning [2], combinatorics [37], and other NP-hard optimization problems [60,62]. Within the context of solvers, algorithm selectors have been proposed for QBF [50,41], SAT [67,68,69], CSP solvers [25,3,34], and recommenders for ATP tools [59,61].

In the setting of SMT solver applications, symbolic execution tools have used algorithm selection strategies [64] and portfolio strategies [33] for the specific classes of instances within the context of the bit-vector theory. This would be an ideal use case of MachSMT, since we provide a more complete solution.

There have been other works using machine learning to improve the performance of SMT solvers. Balunovic et al. [5] use neural networks and synthesis to find tactics and strategies for three SMT-LIB theories. A previous version of our work proposed an algorithm selection tool for the QF_FP theory [57]. To the best of our knowledge, MachSMT is the first publicly available tool for the entirety of SMT-LIB. Other works have leverage machine learning to improve internal heuristics in solvers [12,52,40]

Pairwise ranking has been used in algorithm selection in the latest versions of SATZilla [70], as well as in other settings such as variable selection in the context of splitting heuristics in divide-and-conquer parallel SAT solvers [45].

### 7 Conclusions and Future Work

In this paper, we presented MachSMT, the first algorithm selection tool that spans the entirety of the SMT-LIB logics. MachSMT is designed to be user-friendly and easily modifiable by users for their specific application and SMT solvers of interest.

Using MachSMT, we observe improvement in 54 out of 85 divisions in all tracks from the SMT-COMP 2019 and 2020, with up to a 198.4% improvement for the QF_BVFPLRA SQ '20 division in PAR-2 score. Most of the logics on which we don't see improvement are ones for which we have very few benchmarks.

For future work, we plan to extend our scoring scheme to take into account model validation and unsat core divisions. We further plan to extend our feature set with more (theory-)specific features based on feedback from the SMT community. It is very likely that users may have domain-specific knowledge about certain features that might be most predictive of solver runtime for their particular application. Hence, we have provided an interface to easily extend and specialize MachSMT to a user's specific setting.
